# The Story of the Insane from Year to Year

**Published:** 1895-06-15

**Authors:** 


### THE STORY OF THE INSANE FROM YEAR TO YEAR.

### Eighth Series.

### CITY OF LONDON ASYLUM.

This institution is situated at Stone, near Dartford, in Kent. On January 1st it contained 446 patients, and by the end of the year the numbers had risen to 473. There were 119 admissions, 33 recoveries, 23 discharged relieved, and 29 deaths. The percentage of recoveries on admissions stands at 27·73, and that of the deaths at 6·31 on the average number resident. Both of these percentages are somewhat below the average of kindred institutions; but, as we have before remarked in these notices, a few years' statistics do not prove anything either for or against the asylum. Even statistics covering a long period cannot be relied on in this respect, because the nature of the cases admitted is by far the most important factor in the recovery rate; and then the criterion of sanity has to be known, because what one medical superintendent, of a sanguine nature, would put down as a "recovery"—another, more careful, man would place among the "relieved." Of the deaths, 15 were caused by cerebral and spinal affections, and only 2 by consumption. Turning to the causes of insanity in the year's admissions we find that intemperance in drink is responsible for 25, hereditary influence for 20, religion for 6, and previous attacks for 20. This is one of our public asylums which is doing really good work by admitting private patients at comparatively low rates of payments. At the close of 1894 there were 57 of this class under treatment. Dr. White most truly states that these private patients give more trouble than the paupers, both directly and indirectly, through their friends; but, like other evils of asylum life, this trouble must be borne until such times as the so-called charitable lunatic hospitals open their doors to the reception of this class. As this time is far away in the dim, distant future, it would be a step in the right direction if every county asylum were to build a block for 50 or 100 private patients of the lower middle-class. As mercantile speculations, these blocks would be remunerative, but the good they would do to a large and deserving class is beyond calculation. We did not notice any table showing the average weekly cost per head nor the charges for private patients; but on referring to the blue book for 1893, we find the former was 11s. 8d., and the latter 21s. We are pleased to note that attendant George Hamilton received a pension of £50 a year after 20 years' service.

### CLARE DISTRICT ASYLUM.

The limits of accommodation are given at 380 beds. On January 1st the asylum contained 370 patients, and at the end of the year there were three more in residence. The admissions reached 113, which, considering the size of the asylum, is a high number. Forty were discharged recovered, 13 relieved, and 26 not improved. There were 31 deaths. The recoveries come out at 35·4 per cent. on the admissions, and the deaths at 8·4 on the average number resident. Only four of the deaths were caused by cerebral diseases; general paralysis being absent, as it so often is, from the deaths table in Irish asylums. On the other hand, consumption killed 13, a mortality which would be considered excessive in any English asylum of similar size to Clare. Turning to Table X., which shows the causes of insanity in the year's admissions, we find that 44 were owing to hereditary influence, 7 to drink, 15 to adverse circumstances and domestic trouble, and 3 to religion. In English asylums there is such an antipathy to "restraint" that this means of treatment is often neglected when we are convinced it would be beneficial, and recent legislation will probably make it rarer still. Nevertheless, there cannot be a doubt that mechanical restraint, properly applied and under proper restrictions, is infinitely preferable to that other form of restraint which has not to be recorded in our journals, but which is apt to tire, apt to lose its temper, and is altogether less humane than that kind which it has practically superseded. In Ireland medical superintendents either take a more sensible view of the matter, or they are not trammelled to the same extent as their English brethren. We find that nine patients were restrained in the Clare Asylum during 1894, doubtless with good results to themselves and benefit to the staff; and yet we are convinced there is no more likelihood of a return to the old evil days in Irish asylums than in English ones. The Commissioner says he "found all parts of the asylum clean," and he adds that "Dr. Gelston takes great trouble in looking after the patients' food and creature comforts, and his devotion to the duties of his office deserves commendation." The annual cost of maintenance per head is £21 11s. 11d.

